# Raman Spectroscopy for Assessment of Hard Dental Tissues in Periodontitis Treatment

**DOI:** 10.3390/diagnostics11091595

**Published:** 2021-09-01

**Authors:** Elena V. Timchenko, Irina V. Bazhutova, Oleg O. Frolov, Larisa T. Volova, Pavel E. Timchenko

**Affiliations:** 1 Department of Laser and Biotechnical Systems, Samara National Research University, 443086 Samara, Russia; frolovaleh@gmail.com (O.O.F.); timpavel@mail.ru (P.E.T.); 2 Department of Dentistry, Samara State Medical University, 443079 Samara, Russia; docba@mail.ru; 3 Department Research and Production Center “Samara Tissue Bank”, Samara State Medical University, 443079 Samara, Russia; volovalt@yandex.ru

**Keywords:** Raman spectroscopy, periodontitis, hard dental tissues, closed curettage, professional hygiene

## Abstract

The objective of this work was to use Raman spectroscopy to assess hard dental tissues after professional oral hygiene treatment and curettage. Spectral changes were identified, and the discriminant model of the specific changes of intensity of the Raman lines (i.e., of dentin, cementum, and enamel), before and after the dental procedures, was developed. This model showed that 6 weeks after the procedures, the hard dental tissues did not have differences and, thus, provided similar conditions for bio-film and dental plaque formation, tissue repair, and new attachment to the surface of the root.

## 1. Introduction

In recent years, there has been a sharp increase in the prevalence of periodontal disease. Periodontal disease is a medical and social problem, as periodontitis causes tooth loss, and infections in periodontal pockets could affect one’s body in general [1]. Periodontal inflammatory diseases represent some of the most common dental problems. The prevalence of periodontal inflammatory diseases among the adult population of Russian Federation has reached 80%, and in people over age 65, it is more that 90% [2]. The course of disease is characterized by increasing severity and intensity and the formation of chronic odontogenic center of infection [3]. Chronic catarrhal gingivitis and chronic generalized periodontitis in the early stages are characterized by low-manifestation or a latent period of infection, making it difficult for prompt diagnosis, causing treatment delays; thus, more that 60% of patients seek treatment for moderate periodontitis [4].

The microorganisms of dental calculus play an important role in the multi-factorial etiology of periodontitis; therefore, a clean, smooth surface of a root without bio-films and deposits is the basic requirement for successful periodontal treatment. By doing so, in most cases, the tissues repair and new attachment is formed or connective tissue returns its attachment to the root surface. Therefore, one of the most important stages of treatment is professional oral hygiene, which includes removing above- and under-gum plaque, scaling, and root planning. In some cases, to increase efficiency, the root treatment was supplemented by cutting the inner epithelial lining of the periodontal pocket (the curettage), which is a surgical manipulation with a longer healing time and non-predictable recovery of gum attachment [5,6,7].

The necessity and efficiency of a closed curettage, compared to professional oral hygiene, is still under discussion. The reason is that, the changes in dental tissues after the procedures are not yet fully understood. In the literature, there are opposing views on the necessity of cutting the inner epithelial lining of the periodontal pocket during the root treatment process, so the issue requires further study [5,8].

The obtained results will contribute toward finding additional methods of treatment that could be included in the comprehensive therapy of inflammatory periodontal diseases.

Optical methods are the most wide-spread dental tissue research methods, as they are rapid and non-invasive [9].

Raman spectroscopy is commonly used for medical tasks among the optical methods used in bio-tissue research [10,11]. This method is also used in dentistry.

Mandra et al. [12] used Raman spectroscopy to assess dental tissues with caries. Research shows that this disease causes the reduction of (PO_4_)^3–^ ion concentration.

Gatin et al. [13] studied the processes of bone healing and regeneration in periodontitis treatment using Raman spectroscopy.

Experimental studies using Raman spectroscopy, to assess dental tissues after curettage and professional oral hygiene treatments, did not appear in the literature.

Thus, the objective of this work was to use Raman spectroscopy to assess the hard dental tissues after professional oral hygiene treatment and curettage.

In reference [14], a new technique based on polarized Raman spectroscopy was demonstrated to detect early changes in human enamel caused by artificial caries. The authors showed that, for sound enamel, the Raman peak arising from the symmetric nu1 vibration of PO_4_^3−^ at 959 cm^−1^ was strongly polarized. This is in contrast to the spectra of carious lesions that displayed weaker polarization dependence at 959 cm^−1^. This difference in the degree of the Raman polarization anisotropy allowed for discrimination between early dental caries and sound enamel.

In reference [15], the authors show the efficiency of Raman spectroscopy in determining specific changes in human enamel affected by artificial caries. The results show that Raman maps permit the determination of local variations in the enamel structure affected by the early demineralization process. This investigation contributes to the development of Raman spectroscopy, in the identification of caries at the stage before visual changes in enamel.

In reference [16], the authors show the suitability of Raman spectroscopy, namely by means of the depolarization ratio of the symmetric stretching of the phosphate band as a powerful tool for carious detection (even in early stages). The fluorescence induced by illumination with UV radiation also proved to be a versatile tool for the rapid recognition and delineation of demineralized tissue, and showed promise for differentiation between infected and affected dentine.

## 2. Materials and Methods

A randomized study design was conducted. Fifty-nine premolars removed from European patients, aged 45–62 (average 54.5), of both genders, were used as the subjects of the study. The teeth were removed due to chronic periodontitis. Diagnosis of periodontitis was done clinically; a cone-beam computed tomography (CT) analysis was conducted (the code of the disease, according to ICD-10 (1997)—K05.3).

All samples (the removed teeth) were divided into three main groups: the first group (control group I, 19 teeth)—the teeth diagnosed with chronic periodontitis, removed without prior dental procedures; the second group (study group II, 20 teeth)—the teeth diagnosed with chronic periodontitis, removed six weeks after the professional oral hygiene procedure; the third group (study group III, 20 teeth)—the teeth diagnosed with chronic periodontitis, removed six weeks after the procedure of closed curettage. The samples for the second and the third groups were taken from the same patients; in one side of the jaw, the teeth were professionally cleaned (which included scaling) to remove supragingival and subgingival dental calculus and root planning. On the other side of the jaw, the epithelial lining of the periodontal pocket was cut in addition to the scaling and root planning (thus, the procedure of closed curettage was performed).

The surfaces of the teeth in three different areas were studied: enamel (a), dentin (b) (in longitudinal slices), cementum (c). Three spectra were investigated (with subsequent averaging) in every studied area, at different points of the surface, of every tissue, of each tooth.

Raman spectroscopy was the main method used to assess dental hard tissues before and after the dental procedures. It was implemented using an experimental stand, including a Raman probe RPB-785 (focal length of 7.5 mm) combined with a laser module LML-785.0RB-04 (LuxxMaster Laser, power up to 500 mW, wavelength of 784.7 ± 0.05 nm), and a high-resolution digital spectrometer Shamrock sr-303i providing spectral resolution of 0.15 nm (~1.7 cm^−1^), with a built-in cooling camera DV420A-OE (spectral range of 200–1200 nm) [17].

The spectrum processing was conducted using the software Wolfram Mathematica 12.2. The studied spectrum was cleared from noises via the smoothing median filter (5 points), approximation line (fifth order polynomial) of the spectra fluorescent component was determined in a certain range of 380–1780 cm^−1^, using the iteration algorithm, and this component was subtracted; as a result, the selected Raman spectrum was received.

The detailed analysis of the spectra was made in the software MagicPlotPro 2.7.2 (MagicPlot Systems, LLC) and using the logistic regression method. The Gaussian test function is described by the formula

(1) $$y=a\times e^{-\ln\left(2\right)\times {\left(\frac{x-x_{0}}{dx}\right)}^{2}},$$

where a is normalized amplitude of the line, *dx* is half width at half amplitude (HWHM), *x*_0_ is the line maximum.

The composition of the spectral lines was determined by automatic multi-iteration modeling of the received Raman spectra, in Wolfram Mathematica 12.2, with the use of machine learning methods, and validated by the literature analysis. When modeling the spectral contours at the lines used as a template, the position *x*_0_ and half width of the line (HWHM) *dx* were fixed rigidly. Only the intensities of the lines in the range of 0 to the local maximum of the spectrum at *x*_0_ were selected when modeling. HWHM was limited in the range of 1 to 16 cm^−1^.This allowed us to achieve highly stable results when modeling the contours and to take into account all Raman shifts. The amplitude of the lines *a*, which depended on the values of the independent regressors *dx* and *x*_0_, which define the initial terms of the analysis, was used as a criterion variable.

The average value of a spectrum corrected coefficient of determination for the initial one in the range of 380–1780 cm^−1^ was _adj_R^2^ = 0.9993 for all 176 spectra, which is a good result, but cannot be the only quality criterion.

The normalized amplitudes of the divided Raman lines were used for the relative quantitative analysis of the component composition. The analyses of the received data were conducted using Wolfram Mathematica 12.2, using the logistic regression method.

## 3. Results

**Figure 1:**
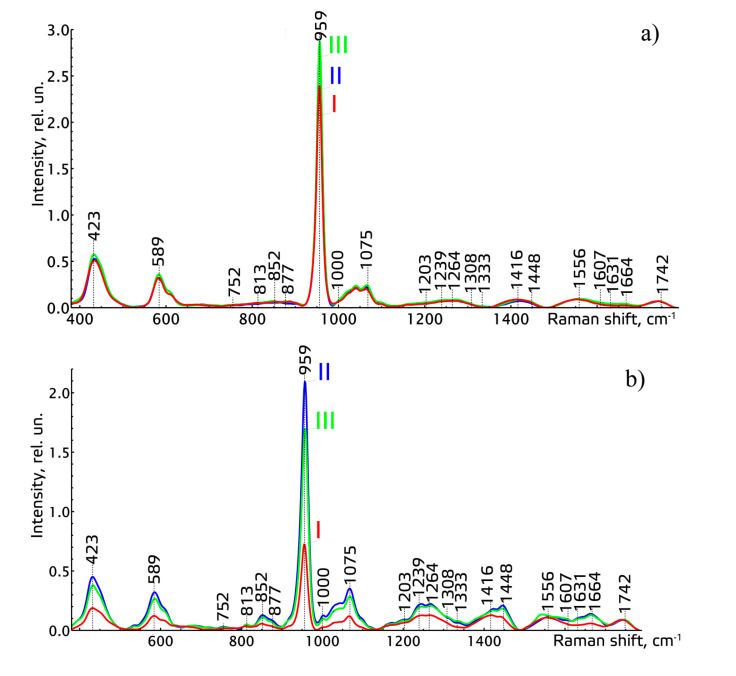

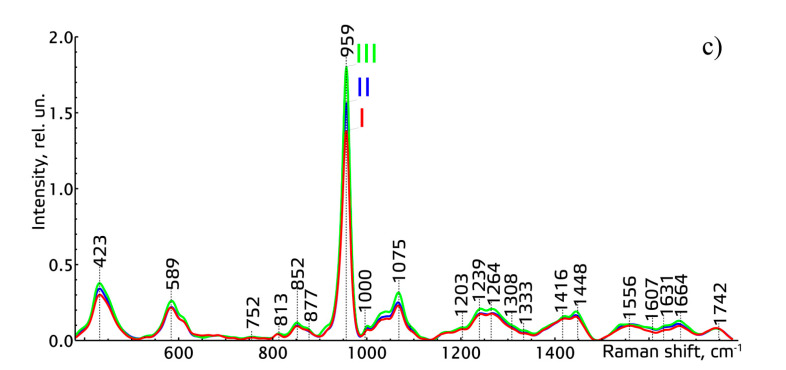
The normalized Raman spectra of enamel (**a**), dentin (**b**), and cementum (**c**) of teeth of the studied groups I–III.

We considered the average normalized Raman spectra of the studied tissues of teeth of the three main groups (Figure 1).

The analysis of the Raman spectra of different tissues of teeth of all the studied groups showed that similar spectral changes (intensity increase of the lines of ~589 cm^−1^, ~423 cm^−1^, ~959 cm^−1^, corresponding to hydroxyapatite, and ~1075 cm^−1^, corresponding to C-O planar valence fluctuation of hydroxyapatite carbonate ion CO_3_^2−^ (ν1)), take place after the procedures of professional oral hygiene and closed curettage, in comparison to the group before the procedures. These spectral changes mainly occurred in the dentin and cementum of teeth. The spectral changes in dentin, after the procedures of scaling and root planning, were caused by penetrating the active mineral components into deeper tissues (dentin) of teeth through special dental tubules [18]. The spectral changes in cementum after the procedures were caused by recovery of mineral components in the tissues and by forming new cementum, due to removing dental calculus and remineralization.

To make the received Raman spectra more informative, a nonlinear regressive analysis of the Raman spectra was conducted, including an investigation of their spectral line decomposition. The whole spectral range of 380–1780 cm^−1^ was divided into four spectral contours: 1–380–508 (_adj_R^2^ = 0.9999), 2–508–1136 (_adj_R^2^ = 0.9978), 3–1136–1491 (_adj_R^2^ = 0.9998), 4–1491–1781 cm^−1^ (_adj_R^2^ = 0.9998).

**Figure 2:**
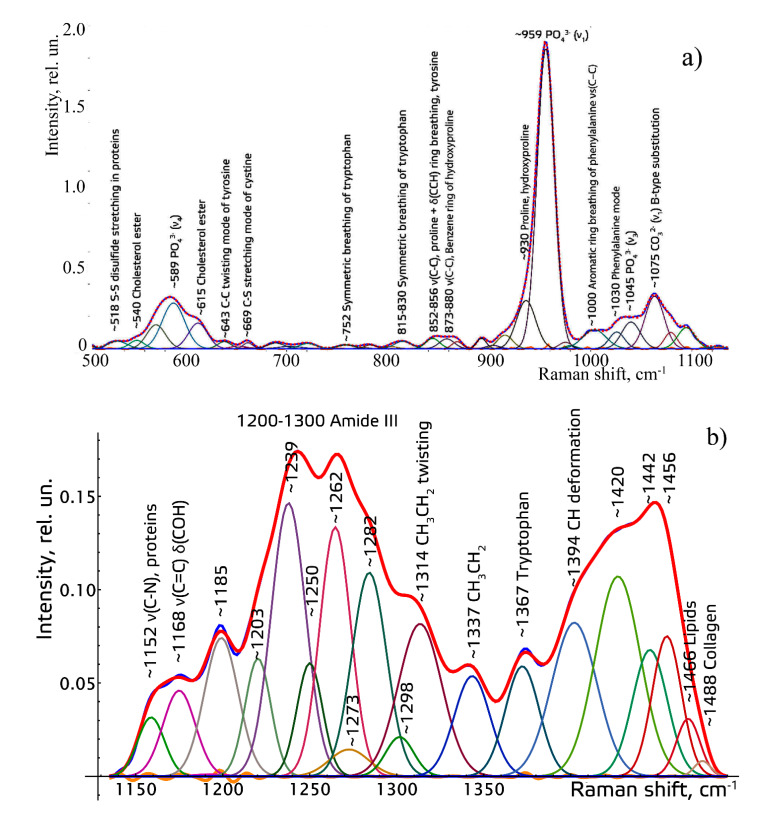

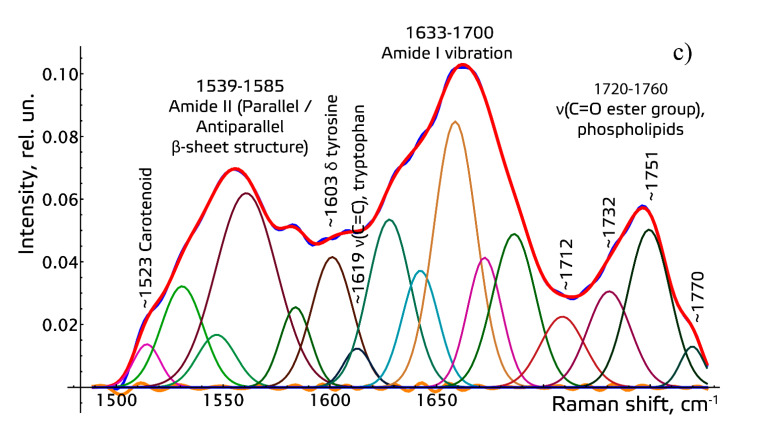
The example of decomposition of spectral contours of the studied dental tissues: (**a**) in the range of 508–1136 cm^−1^, (**b**) in the range of 1137–1490 cm^−1^, (**c**) in the range of 1491–1781 cm^−1^.

Figure 2 shows the results of decomposition of the spectral contours on the sum of distribution of the Gaussian lines.

**Figure 3:**
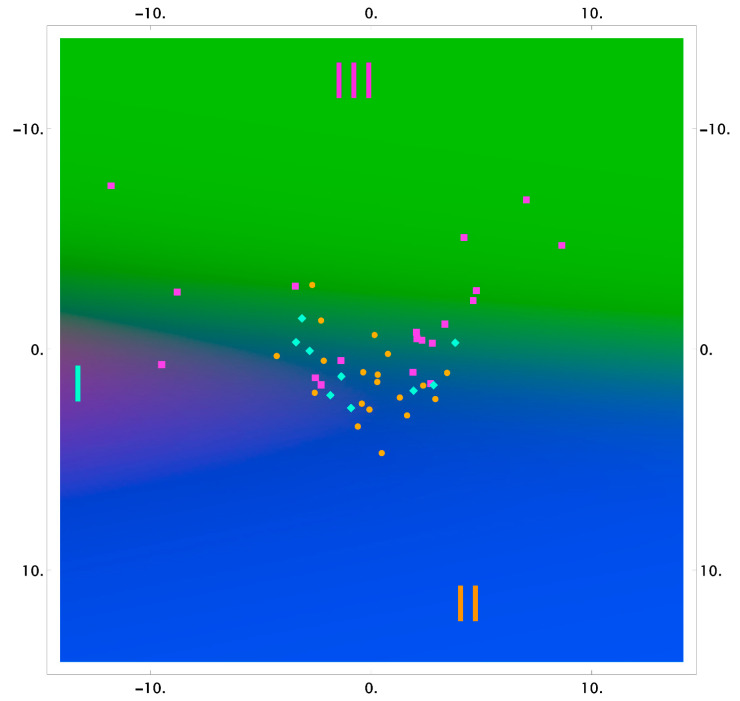
The 2D probability distribution of each class as a function of the features (enamel) of the studied groups I–III.

**Figure 4:**
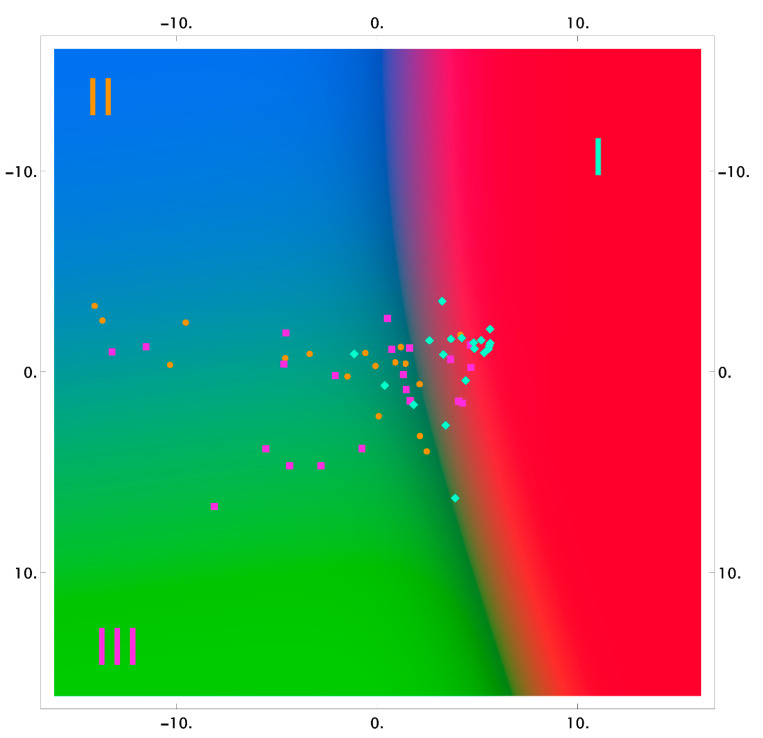
The 2D probability distribution of each class as a function of the features (dentin) of the studied groups I–III.

**Figure 5:**
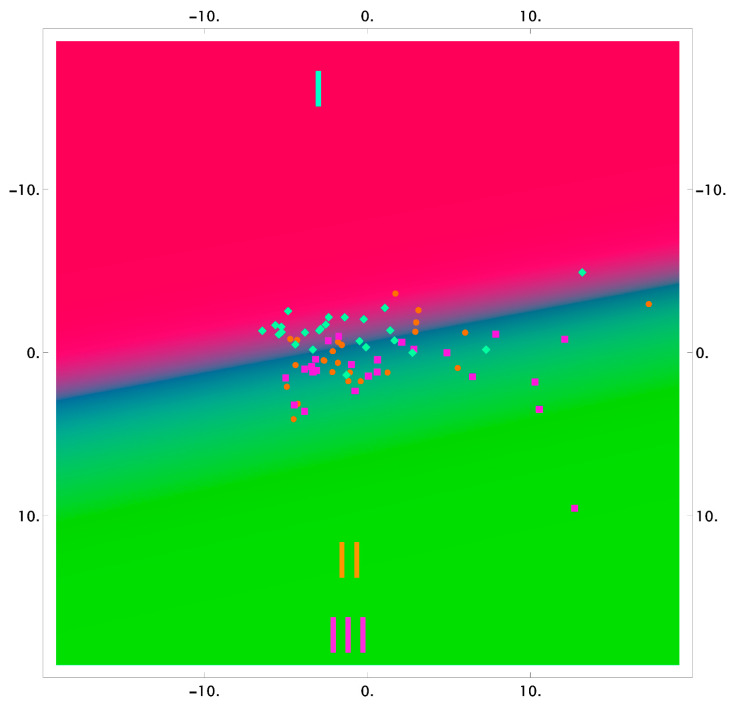
The 2D probability distribution of each class as a function of the features (cementum) of the studied groups I–III.

The results of the classification using the logistic regression method, in reduced two-dimensional measurements, are shown in Figure 3, Figure 4 and Figure 5 as the probability distribution of each measurement, classified as one of the three studied groups.

Figure 3, Figure 4 and Figure 5 show that the areas of the measurements of the group of samples taken after the dental procedures (professional oral hygiene and closed curettage) significantly intersect and do not allow highlighting differences of spectral composition between the groups. However, there are differences between these groups, and the group of samples taken before dental procedures (red area) in tooth dentin and cementum.

**Figure 6:**
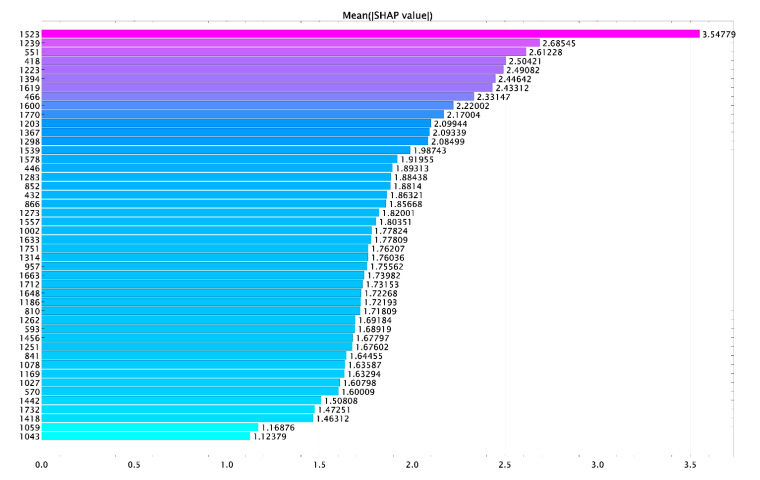
Mean (|SHAP values|).

Figure 6 shows the influence of the variables (Raman lines) on the result of classification of cementum tissues.

The analysis using the logistic regression method allowed building a discriminative model of the three groups of intensity-specific changes of the dentin, cementum, and enamel Raman lines. The correctly classified changes in the samples with periodontitis were 93% with cross-validation. The calculated values for the group of samples with periodontitis were recall = 100%, precision = 86.1%, specificity = 94.8%, AUC = 98.5%.

The resulting verification of accuracy of the model was performed using an extra 44 randomly selected measurements that we did not use for the analysis. The model allows classifying the samples with periodontitis with an accuracy of 93%, the samples having professional oral hygiene with an accuracy of 47%, and the samples after the closed curettage with an accuracy of 33%. The received data indicate no statistically significant changes in the Raman spectra 6 weeks after the closed curettage as compared to professional oral hygiene.

## 4. Discussion

Despite the existing progress in the study of periodontitis [19,20,21,22], our study results are of great importance for the diagnosis and treatment of periodontal diseases, could help significantly improve the quality of life of patients during therapy, and shorten the rehabilitation period.

Our previously published work [22] contained diagnosed spectral changes in the tissues of teeth with severe periodontitis and the developed algorithm of verification of tooth enamel with periodontitis to identify at-risk patients and perform diagnostic screening of patients with periodontitis.

This work is an extension of our previous research, and aims to assess and correct the main methods of periodontitis treatment (professional oral hygiene and closed curettage) to reveal their efficiency, with the objective to reduce invasiveness and recovery time after treatment.

The significance of changes of dental tissues after dental procedures (periodontitis treatment) is still not fully understood in other works. The authors of [23,24,25,26,27] showed that the main structural changes occurred in cementum.

The earliest works of the authors of [23] showed that wrong formation of cementum caused periodontal pockets. The reason was that cementum structure was the main element of microbial infestation susceptibility and susceptibility to forming periodontal pockets.

Later, in [24], comparative analysis of two groups of patients diagnosed with periodontitis and gingivitis was made. The comparison showed that mineral composition of cementum of teeth with chronic periodontitis was higher than with gingivitis. The authors explained it as oral fluid influencing the roots, causing mineral deposition on the surface of tooth cementum.

The authors of [25,26,27] revealed that the reasons for attachment loss during aggressive periodontitis were bacteria and cementopathia. The authors showed the changes of form and structure of tooth cementum with different types of periodontitis, which were different from gingivitis. The analysis of these works showed that one of the reasons of periodontitis is cementum defects. This means that the changes of cementum structure can be considered as risk factors for periodontitis and, therefore, the tooth cementum restoration using dental procedures can treat this disease.

The experimental studies we made allowed establishing that the main spectral changes after the dental procedures (after deep cleaning and closed curettage) occurred both in the inner structures of teeth (in dentin) and in the outer structures (in cementum_. The spectral changes in tooth cementum and dentin after dental procedures were identified; these changes increased the intensity of the lines ~589 cm^−1^, ~423 cm^−1^, ~959 cm^−1^, corresponding to hydroxyapatite, and ~1075 cm^−1^ corresponding to C-O planar valence fluctuation of hydroxyapatite carbonate ion CO_3_^2−^(ν_1_).

The spectral changes in tooth dentin after dental procedures, most likely, were caused by penetrating and deposits of mineral components through spaces between the layers of cementum and exposing the dentinal tubules during periodontitis. The changes of dentin and cementum mineralization during periodontitis were described in [28].

The changes in tooth cementum were caused by forming new cementum due to removing dental calculus and remineralization.

Animal studies, proving that periodontal tissue recovery depends on the condition of the tooth surface, are described in [29,30,31,32,33].

This work identifies the spectral changes in the tissues of teeth before and after the main stages of periodontitis treatment (professional oral hygiene and closed curettage), the developed discriminative model of the specific changes of intensity of the Raman lines of the studied tissues of teeth before and after the dental procedures, which showed that 6 weeks after the professional oral hygiene and closed curettage, the hard dental tissues did not have differences and, thus, provided similar conditions for bio-film and dental plaque formation, tissue repair, and new attachment to the surface of a root.

## 5. Conclusions

The possibility of using the Raman spectroscopy for noninvasive rapid assessment of efficiency of periodontitis treatment, based on changes in the tooth cementum spectra, was shown.

The results of this research will allow to correct the treatment of patients with periodontitis and to avoid non-effective stages of comprehensive treatment of inflammatory periodontal disease in dental practice.
